# Efficacy and safety of rechallenge [^177^Lu]Lu-PSMA-617 RLT after initial partial remission in patients with mCRPC: evaluation of a prospective registry (REALITY study)

**DOI:** 10.1007/s00259-024-06825-4

**Published:** 2024-07-15

**Authors:** Florian Rosar, Joelle Schuler, Caroline Burgard, Arne Blickle, Mark Bartholomä, Stephan Maus, Sven Petto, Fadi Khreish, Andrea Schaefer, Samer Ezziddin

**Affiliations:** 1 Department of Nuclear Medicine, Saarland University – Medical Center, Kirrberger Str. 100, Geb. 50, D-66421 Homburg, Germany; 2 Department of Nuclear Medicine, Campus-Fulda, University of Marburg, Fulda, Germany

**Keywords:** Rechallenge, PSMA, Radioligand therapy, ^177^Lu, mCRPC

## Abstract

**Aim:**

Rechallenge of [^177^Lu]Lu-PSMA-617 radioligand therapy (RLT) was proposed for patients who initially responded to PSMA-RLT experiencing partial remission, but relapsed into progression after a certain period of remission. However, only limited data is available regarding this approach. In this study, we analyzed the efficacy and safety profile of one or more series of [^177^Lu]Lu-PSMA-617 RLT rechallenge in patients from a prospective registry (REALITY Study, NCT 04833517) after they initially benefited from PSMA-RLT.

**Methods:**

Forty-seven patients with metastatic castration-resistant prostate cancer (mCRPC) who had biochemical response to initial [^177^Lu]Lu-PSMA-617 RLT followed by disease progression received at least one (up to three) series of [^177^Lu]Lu-PSMA-617 RLT rechallenge. Biochemical response rates based on prostate-specific antigen (PSA) serum value, PSA-based progression-free survival (PFS) and overall survival (OS) were calculated. Adverse events of the treatment were assessed according to ‘*common terminology criteria for adverse events*’ (CTCAE).

**Results:**

After one series of RLT rechallenge, a PSA decline of at least 50% was achieved in 27/47 patients (57.4%). The median PFS of all patients was 8.7 mo and the median OS was 22.7 mo, each calculated from the administration of the first rechallenge series. Patients who responded (PSA decline > 50%) to the rechallenge showed a median OS of 27.3 mo. Regarding PFS, a significant correlation (*r* = 0.4128, *p* = 0.0323) was found for these patients comparing initial and rechallenge RLT. Ten patients received a second and 3 patients received a third rechallenge series with 8/10 and 3/3 patients responding to repeated RLT rechallenge. No severe deterioration of adverse events rated by CTCAE criteria was observed.

**Conclusion:**

[^177^Lu]Lu-PSMA-617 RLT rechallenge is associated with significant PSA response and encouraging survival outcome as well as a very favourable safety profile and should therefore be considered as a straight-forward treatment option in mCRPC patients, who previously benefited from PSMA-RLT.

**Supplementary Information:**

The online version contains supplementary material available at 10.1007/s00259-024-06825-4.

## Background

Prostate cancer remains one of the most prevalent forms of malignant diseases in men worldwide in 2022 [1]. Patients with prostate cancer frequently progress into the advanced stage of metastatic castration-resistant prostate cancer (mCRPC) which is resistant to physical or chemical castration by androgen deprivation therapy (ADT) and is attributed with a poor prognosis [2–4]. In this scenario treatments with androgen receptor signaling inhibitor (ARSI) [5, 6], taxane-based chemotherapy [7, 8], ^223^Ra treatment [9] or PARP-inhibitors [10, 11] are established treatment options. In recent years, radioligand therapy (RLT) targeting prostate-specific membrane antigen (PSMA) has been demonstrated to be effective, safe and well-tolerated in several retrospective and prospective studies on mCRPC [12–18]. Based on the phase 3 VISION trial PSMA-RLT with ^177^Lu was finally approved by both the FDA and EMA [17, 19, 20]. However, patients who initially responded excellently to PSMA-RLT and showed a temporary period of remission tend to relapse into progression. In this rather late stage of disease, only limited treatment options for patients remain. Rechallenge of PSMA-RLT might be a reasonable approach, particularly in patients who previously had an excellent response to this treatment. However, data on this topic are scarce and only reported for a limited number of patients [21–23]. In this study, we analyzed the efficacy and safety profile of one or more rechallenge series of [^177^Lu]Lu-PSMA-617 RLT in patients from a prospective registry (REALITY Study, NCT 04833517) who initially responded to PSMA-RLT and experienced partial remission.

## Materials and methods

### Study design and patient population

**Fig. 1:**
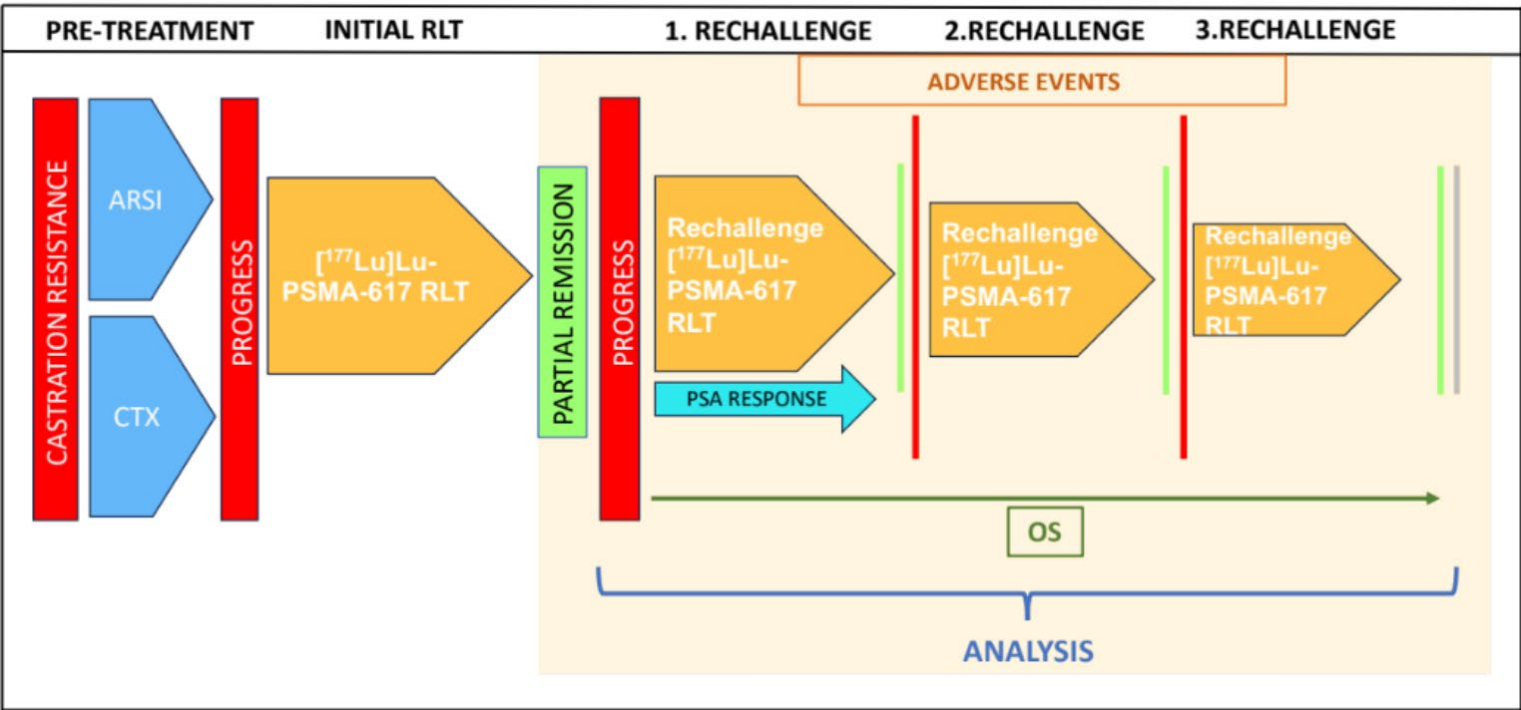
Study design

The aim of this study was to evaluate the clinical value of [^177^Lu]Lu-PSMA-617 RLT rechallenge in mCRPC. Rechallenge was defined as the retreatment with one or more series of [^177^Lu]Lu-PSMA-617 (each series comprising multiple sequential treatment cycles) in patients who responded to the initial [^177^Lu]Lu-PSMA-617 series, followed by a relevant progression-free time period and renewed progression. Response was characterized as a decrease in prostate-specific antigen (PSA) serum value ≥ 50% during treatment, whereas an increase of PSA value ≥ 25% was assessed as progression. After progression occurred, the patients received a rechallenge series of [^177^Lu]Lu-PSMA-617 RLT. The primary endpoint comprised evaluation of PSA response rate and outcome. The secondary endpoint included the analysis of adverse events. The study design is displayed in Fig. 1.

**Fig. 2:**
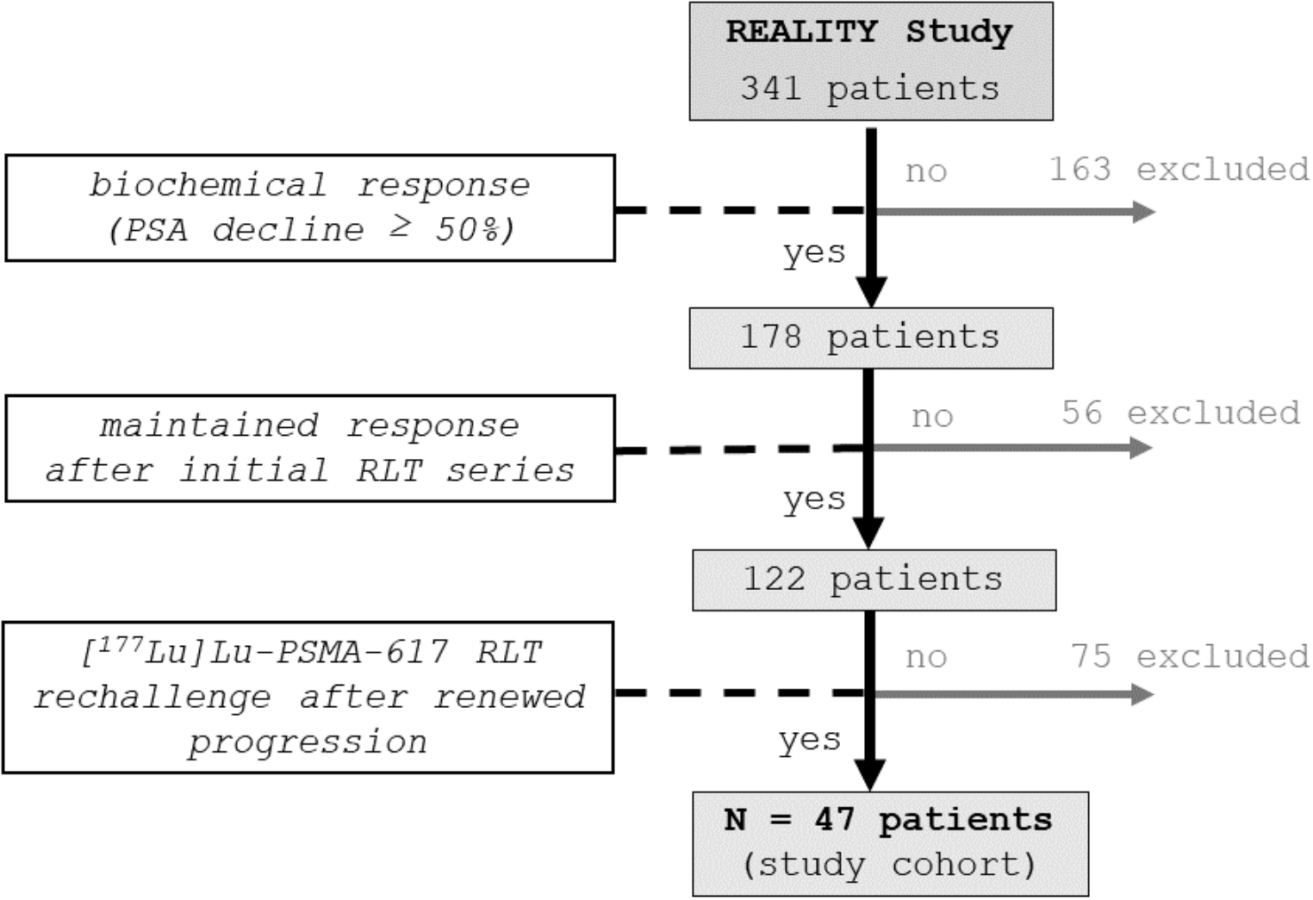
Flow chart of study patient selection

All patients were enrolled in the ‘*prospective registry to assess outcome and toxicity of targeted radionuclide therapy in patients with mCRPC in clinical routine’ (REALITY Study*; NCT04833517*)* between January 2016 and September 2022. In this time frame, 341 mCRPC patients were treated with [^177^Lu]Lu-PSMA-617 RLT within this registry. In total, *n* = 47 patients of the initial cohort could be identified fulfilling the following inclusion criteria (Fig. 2): (I) biochemical response to the initial [^177^Lu]Lu-PSMA-617 RLT series; (II) maintained response after initial RLT series; (III) [^177^Lu]Lu-PSMA-617 RLT rechallenge after relevant progression-free time period and renewed progression. Accordingly, 163/341 patients were excluded due to missing biochemical response during the initial series of [^177^Lu]Lu-PSMA-617 RLT. Fifty-six of these 178 patients were subsequently excluded because after temporarily responding but not maintaining response up to the end of the initial series. Out of the remaining 122 patients, 47 received [^177^Lu]Lu-PSMA-617 RLT rechallenge after relevant progression-free time period and renewed progression and were finally included in the analysis. All patients of the cohort were heavily pretreated with androgen-deprivation therapy (ADT), androgen-receptor signaling inhibitors (ARSI) and/or chemotherapy. Detailed patient characteristics are compiled in Table 1.

**Table 1:** *Patient characteristics*

Characteristic	Value
*Age*	
Median (range) in [years]	72 (58–87)
*ALP*	
Median (range) in [U/L]	94 (45–241)
*Hemoglobin*	
Median (range) in [U/L]	12.3 (7.6–16.1)
< 13 g/dL, *n* (%)	32 (68)
*ECOG performance status*, *n* (%)	
0	19 (40.4)
1	21(44.7)
≥2	7 (15)
*PSA*	
Start of initial RLT, median (range) in [ng/mL]	100 (1.5–9579)
Start of rechallenge RLT, median (range) in [ng/mL]	103 (1.0–5475)
*Sites of metastases*, *n* (%)	
Bone	43 (91.5)
Lymph node	30 (63.8)
Liver	7 (25)
Other	20 (42.5)
*Prior therapies*, *n* (%)	
Prostatectomy	21 (44.7)
Radiation	28 (59.6)
ADT	47 (100)
ARSI	
Abiraterone	35 (74.5)
Enzalutamide	36 (76.6)
Abiraterone + Enzalutamide	27 (57.4)
Chemotherapy	
Docetaxel	28 (59.6)
2nd line Cabazitaxel	14 (29.8)
[^223^Ra]Ra-dichloride	7 (15)
*Initial RLT series*	
Number of cycles, median (range)	3 (1–8)
^177^Lu activity, median (range) in [GBq]	
Per cycle	6.2 (4.3–9.1)
Cumulative	20.3 (6.9–48.6)
*First rechallenge series*	
Patients, n (%)	47 (100)
Number of cycles, median (range)	3(1–6)
^177^Lu activity, median (range) in [GBq]	
Per cycle	7 (4.3–9.3)
Cumulative	17.2 (4.4–44.3)
*Second rechallenge series*	
Patients, n (%)	10 (21.3)
Number of cycles, median (range)	2 (1–5)
^177^Lu activity, median (range) in [GBq]	
Per cycle	7,63 (4.4–9.1)
Cumulative	15.5 (9.1–30.5)
*Third rechallenge series*	
Patients, n (%)	3 (6.4)
Number of cycles, median (range)	2 (2–3)
^177^Lu activity, median (range) in [GBq]	
Per cycle	7.1 (4.3–9.1)
Cumulative	15.9 (8.6–23.6)
*Overall*	
Number of cycles, median(range)	7 (2–17)
Cumulative ^177^Lu activity, median (range) in [GBq]	66.3 (12.4–118.8)

ADT: androgen deprivation therapy; ALP: alkaline phosphatase; ECOG: eastern cooperative oncology group; ARSI: androgen-receptor signaling inhibitors; PSA: prostate-specific antigen

The initial series of PSMA-RLT encompassed administration of a median of 3 cycles (range: 1–8 cycles) with a median administered activity of 6.20 GBq/cycle (range: 4.33–9.10 GBq/cycle) [^177^Lu]Lu-PSMA-617. Detailed information on the initial PSMA-RLT series is provided in Table S1 in the supplemental material. The initial series of PSMA-RLT was discontinued when patients experienced remarkable biochemical response with only limited tumor load remaining. The mean PSA decline observed after the initial series of PSMA-RLT was 85.5 ± 14.4% (median: 91.5%; range: 53–99%). The rechallenge, i.e. the second [^177^Lu]Lu-PSMA-617 RLT series was initiated after a PSA-based progression-free survival (PFS) of median 10.8 months. Sufficient PSMA expression was verified by PSMA-targeted positron emission tomography/ computed tomography (PET/CT) and defined as markedly higher tumoral tracer uptake compared to the healthy liver. The administration of [^177^Lu]Lu-PSMA-617 RLT was applied on a compassionate use basis, following the regulations of the German Pharmaceutical Act § 13 (2b). The protocol of this study was in accordance with the declaration of Helsinki. All patients provided written consent after being informed about the general risks and potential negative side effects of this treatment and agreed to the publication of any data resulting from the study in anonymized form. The study was approved by the local institutional review board (ethics committee permission number 140/17).

### Treatment details of PSMA-RLT rechallenge

The radiolabeling of PSMA-617 with ^177^Lu and the quality control of [^177^Lu]Lu-PSMA-617 were accomplished based on the standard procedures described by Kratochwil et al. [24]. The radionuclide ^177^Lu was provided by IDB Holland BV (Baarle-Nassau, Netherlands) and PSMA-617 by ABX advanced biochemical compounds GmbH (Radeberg, Germany).

Administered activities were adjusted to the characteristics of each individual patient considering tumor burden, location of metastases, diffuse involvement of bone marrow, course of disease, general physical condition, body weight, body surface, renal function, and blood cell count. 30 min prior to infusion of [^177^Lu]Lu-PSMA-617, the patient received hydration by 500 mL 0.9% NaCl intravenously and additionally a cooling of the salivary glands was applied. Infusion of [^177^Lu]Lu-PSMA-617 was administered over a period of one hour.

All 47 patients received a rechallenge, i.e. a second series of [^177^Lu]Lu-PSMA-617 RLT comprising a median of 2 cycles (range: 1–6 cycles) with a median administered activity of 7.0 GBq/cycle (range 4.25–9.25 GBq/cycle). A second rechallenge i.e. a third series of [^177^Lu]Lu-PSMA-617 RLT applied to 10 patients, comprised a median of 2 cycles (range: 1–5 cycles) with a median administered activity of 7.63 GBq/cycle (range: 4.4–9.1 GBq/cycle). Three patients of the cohort also received a third rechallenge i.e. a fourth series of [^177^Lu]Lu-PSMA-617 consisting of a median of 2 cycles (range: 2–3 cycles) administering a median activity of 7.9 GBq/cycle (range: 4.5–9.3 GBq/cycle). The mean interval between the second and third series and between the third and fourth series was 9.7 ± 5.4 months and 15.1 ± 3.0 months, respectively.

PSMA-RLT rechallenge series were discontinued when patients experienced remarkable biochemical response with only limited residual tumor burden (depicted by post-therapeutic [^177^Lu]Lu-PSMA-617 scintigraphy or [^68^Ga]Ga-PSMA-11 PET/CT) or when progression was observed.

### Response evaluation and outcome

Response was biochemically assessed by measurement of PSA serum value during and after each series of [^177^Lu]Lu-PSMA-617 RLT. Following the recommendations of the prostate cancer working group 3 (PCWG3) [25] an increase of PSA value ≥ 25% was defined as progressive disease (PD). A decrease of PSA ≥ 50% was rated as partial remission (PR), while a change of PSA ranging from + 25% to -50% was rated as stable disease (SD). Patients who experienced a PR were characterized as responders, and patients with SD or PD as non-responders.

PSA-based progression-free survival (PFS) was calculated specifically for each rechallenge with the start of the corresponding series and the endpoint of either biochemical PD or last study visit. Overall survival (OS) was calculated starting at the date of initiating the second PSMA-RLT series (first rechallenge) and ending either at the occurrence of death from any cause or last contact. The cut-off date of the study was 31st October 2023. All statistical analyses were performed using PRISM 9 software (GraphPad Software, San Diego, USA). Level of significance was defined as *p*-value < 0.05. PFS and OS were determined using the Kaplan-Meier method (log-rank test). Spearman correlation was used to calculate the relation between PFS of the initial series and the first rechallenge series.

### Recording of adverse events

To assess adverse events during and after the RLT series the ‘*common terminology criteria for adverse events*’ (CTCAE), version 5.0 (https://ctep.cancer.gov/protocoldevelopment/electronic_applications/docs/CTCAE_v5_Quick_Reference_5x7.pdf; last accessed 7th May 2024), were used. Renal impairment, anemia, thrombocytopenia, leukopenia, fatigue and xerostomia were analyzed. While anemia, thrombocytopenia, leukopenia and renal impairment were evaluated by frequent blood cell count and glomerular filtration rate (GFR), xerostomia and fatigue were assessed by using a questionnaire following CTCAE terminology.

## Results

### PSA response

**Fig. 3:**
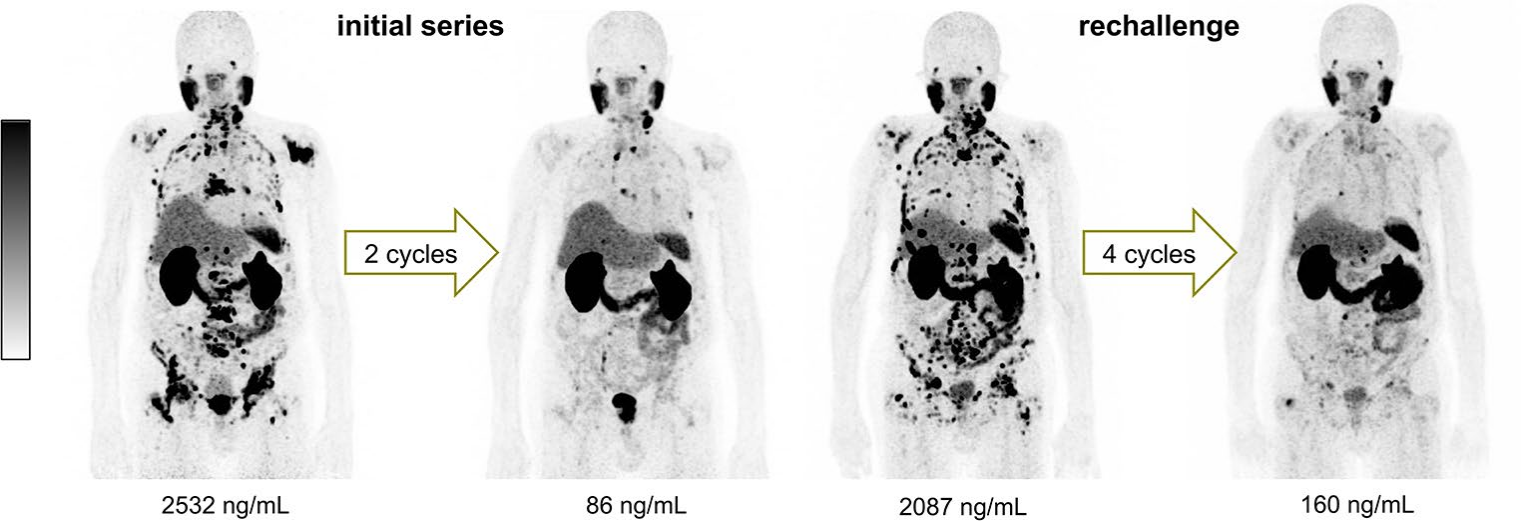
Maximum intensity projection of [^68^Ga]Ga-PSMA-11 PET/CT images of a representative patient with biochemical and molecular imaging response after the initial series (2 cycles) and the rechallenge series (4 cycles) of [^177^Lu]Lu-PSMA-617 (time interval between the initial series and the rechallenge: 3.25 years)

Preceding the administration of the first [^177^Lu]Lu-PSMA-617 RLT rechallenge a mean serum PSA value of 346 ± 845 ng/mL (range: 1.00–5475 ng/mL) was observed, which decreased to 75 ± 112 ng/mL (range 1.00–663 ng/mL) under therapy, implying a mean PSA decline of 46.6% ± 47.1%. In total, 27/47 patients (57.4%) experienced partial remission (PR), 17/47 (36.2%) stable disease (SD) and 3/47 (6.4%) progressive disease (PD). A representative example of a patient with PR after rechallenge RLT is presented in Fig. 3.

**Fig. 4:**
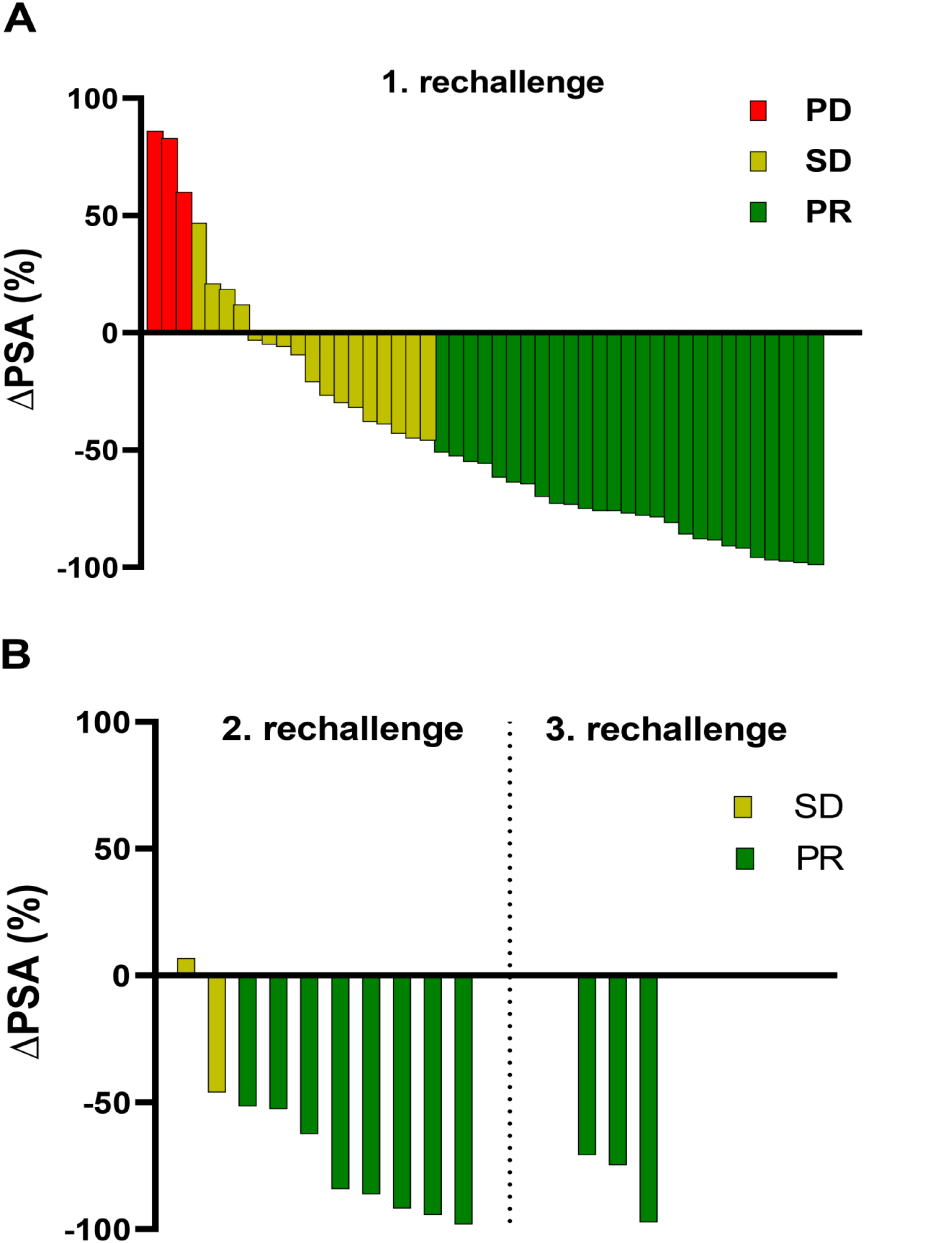
Waterfall-plots representing the individual change of PSA value (ΔPSA) after (**A**) the first and (**B**) the second and the third rechallenge of [^177^Lu]Lu-PSMA-617 with categorization into progressive disease (PD), stable disease (SD) or partial remission (PR)

**Fig. 5:**
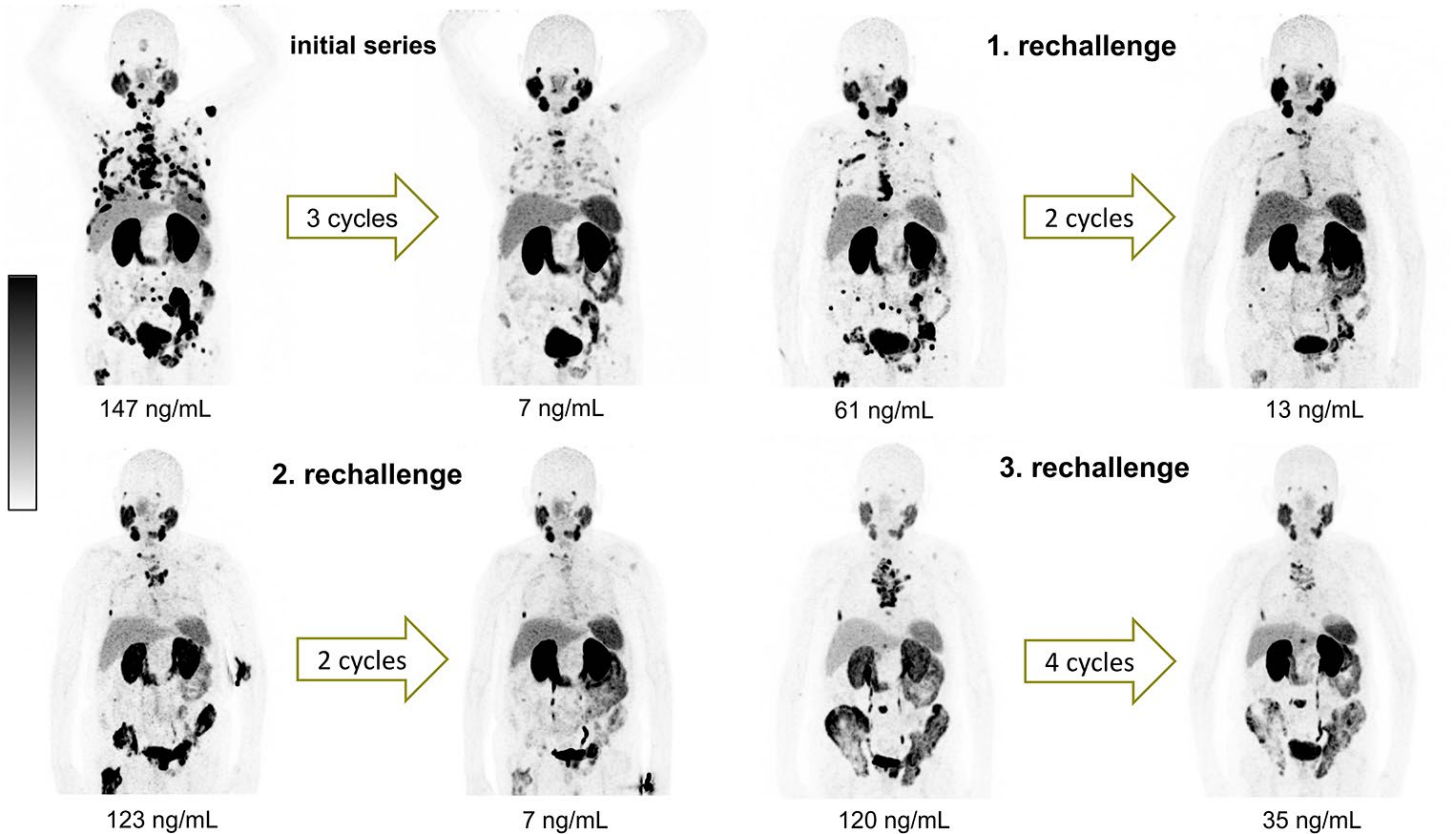
Maximum intensity projection of [^68^Ga]Ga-PSMA-11 PET/CT images of a representative patient with biochemical and molecular imaging response after initial series and 3 rechallenge series of [^177^Lu]Lu-PSMA-617

For patients who underwent a second rechallenge series of [^177^Lu]Lu-PSMA-617 RLT, the mean PSA value decreased by 67.4% ± 29.0%, from 203 ± 213 ng/mL (range: 49–826 ng/mL) to 93 ± 109 ng/mL (range 1.40–385 ng/mL) with PR, SD and PD in 8/10 (80%), 2/10 (20%) and 0/10 (0%) patients, respectively. During a third rechallenge series, applied to 3 patients, a mean PSA value of 379 ± 281 ng/mL (range: 120–678 ng/mL) at start and 72 ± 87 (range: 9.50–171 ng/mL) after completion of the treatment series was observed. All patients (3/3, 100%) showed a PR with a mean PSA decline of 80.9% ± 14.2%. Figure 4 represents a waterfall plot of the individual PSA changes for all rechallenge series. Figure 5 exemplarily shows a patient who received a baseline [^177^Lu]Lu-PSMA-617 RLT followed by three rechallenge series. Each treatment series induced PR characterized by remarkable PSA decline.

### Outcome

**Fig. 6:**
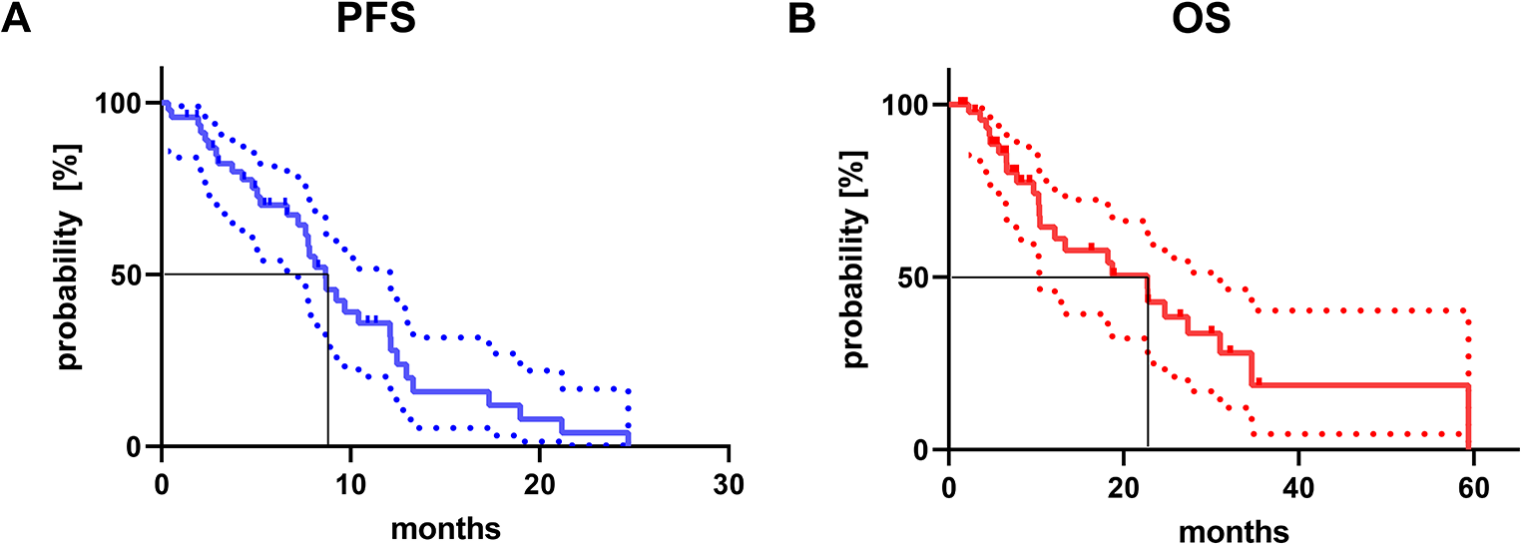
Kaplan-Meier curves presenting (**A**) PSA-based progression-free survival (PSF) for the first rechallenge series and (**B**) overall survival (OS), both calculated from the start of rechallenge RLT

**Fig. 7:**
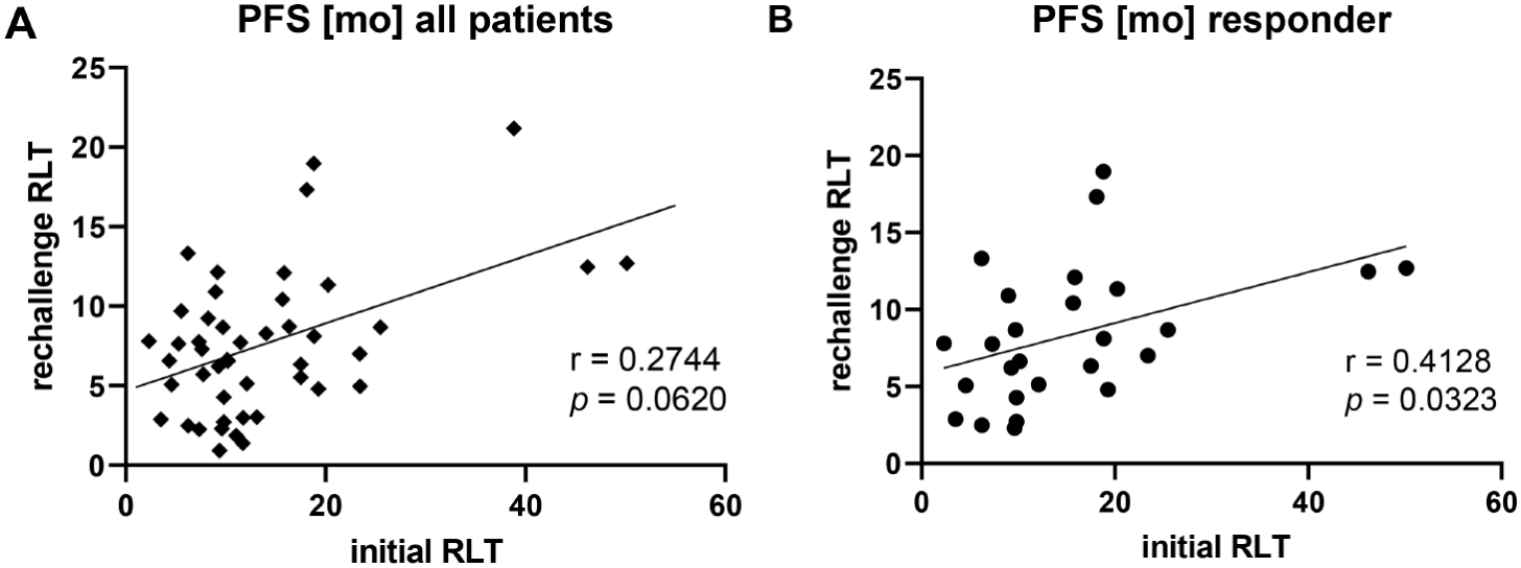
Progression-free survival after initial series vs. first rechallenge series of [^177^Lu]Lu-PSMA-617 for (**A**) the total patient cohort, depicting no significant correlation between the two series and for (**B**) responders to rechallenge RLT, demonstrating a significant correlation between the series

The median PSA-based progression-free survival (PFS) and the overall survival (OS), both calculated from the administration of the first rechallenge cycle with [^177^Lu]Lu-PSMA-617, were analyzed. As depicted in Fig. 6A the median PFS was 8.7 months (CI: 0.5–39.2 months). Data regarding OS displayed in Fig. 6B, showed a median of 22.7 months (CI: 18.2–24.7 months) after administration of the first rechallenge series. While patients who responded to the rechallenge approach, i.e. experienced a PR, showed a median OS of 27.3 months (CI: 16.3–34.6 months), patients with SD or PD showed a significantly shorter (Log-rank test *p* = 0.0302) median OS of 10.3 months (CI: 8.3–22.7 months). If considering the initial therapy with [^177^Lu]Lu-PSMA-617 RLT as the starting point for OS calculation, the median OS of the patients was 18.7 months (CI: 10.6–78.4 months). Regarding PFS no significant correlation was observed comparing initial and rechallenge RLT for all patients (*r* = 0.2744, *p* = 0.0620, Fig. 7A). However, in a subgroup analysis including responders to rechallenge, a moderate significant positive correlation was found (*r* = 0.4162, *p* = 0.0323, Fig. 7B).

### Adverse events

**Fig. 8:**
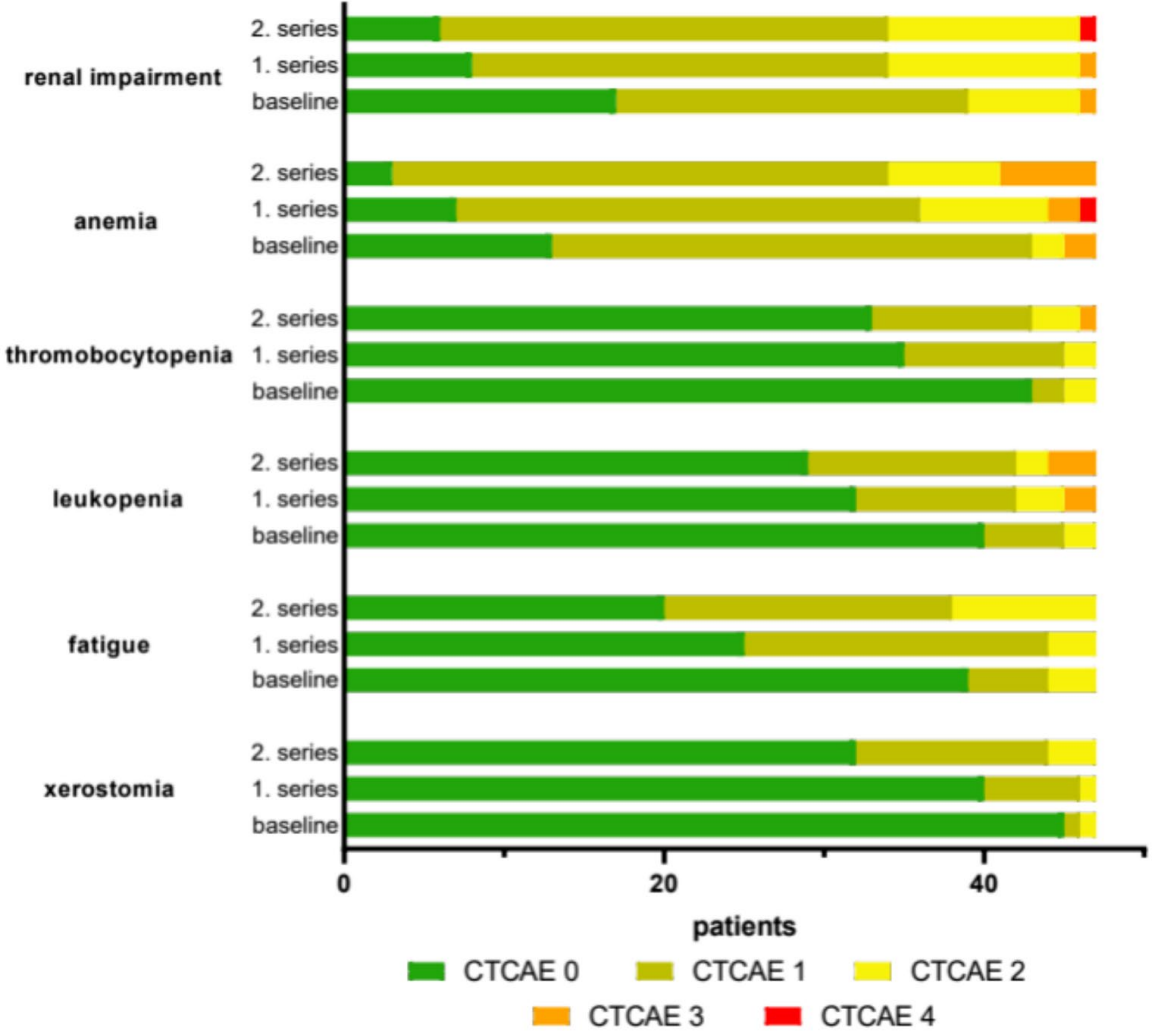
Bar diagram presenting adverse events categorized according to the ‘common terminology criteria for adverse events’ (CTCAE), each apportioned for pre-RLT (baseline), the initial series (1. series) and the first rechallenge (2. series) of [^177^Lu]Lu-PSMA-617

The majority of recorded events was categorized as mild or moderate (CTCAE score 1 or 2). Figure 8; Table 2 present the documented adverse events, based on the CTCAE terminology, for the initial series, first and second rechallenge of RLT. A slight increase in CTCAE grade 1/2 events was observed comparing first and second rechallenge treatment series (17.0% for xerostomia, 10.6% for fatigue, 4.3% for leukopenia, 2.1% for thrombocytopenia, 1.7% for anemia and 4.3% for renal impairment). CTCAE grade 3/4 events were rarely experienced. The comparison of initial and rechallenge RLT revealed a minor increase for leukopenia (2.1%), thrombocytopenia (2.1%) and anemia (6.4%), while no change for renal impairment and no case of CTCAE grade 3/4 for xerostomia and fatigue was recorded. To summarize, the frequency and severity of all treatment-related adverse events occurring for patients who received two (*N* = 10) or three (*N* = 3) rechallenge series a respective table (Table S2) is presented in the supplemental material. In addition, Figure S1 of the supplemental material shows the individual course of GFR in these patients over the initial and the rechallenge series of RLT.

**Table 2:** Patient-based incidence and severity of adverse events observed before starting [^177^Lu]Lu-PSMA-617 RLT and over the course of initial series and first rechallenge series of RLT in 47 patients with mCRPC

**AE**	Total	Total		Grade 1/2		Grade 3/4	
		n	%	n	%	n	%
Any	pre-RLT	43	91.50%	40	85.10%	3	6.40%
	initial RLT	44	93.60%	39	83.00%	5	10.60%
	1st Rechallenge RLT	46	97.90%	41	87.20%	5	10.60%
Xerostomia	pre-RLT	2	4.30%	2	4.30%	0	0
	initial RLT	7	14.90%	7	14.90%	0	0
	1st Rechallenge RLT	15	31.90%	15	31.90%	0	0
Fatigue	pre-RLT	8	17.00%	8	17.00%	0	0
	initial RLT	22	46.80%	22	46.80%	0	0
	1st Rechallenge RLT	27	57.50%	27	57.50%	0	0
Leukopenia	pre-RLT	7	14.90%	7	14.90%	0	0
	initial RLT	15	31.90%	13	27.60%	2	4.30%
	1st Rechallenge RLT	18	38.30%	15	31.90%	3	6.40%
Thrombocytopenia	pre-RLT	4	8.50%	4	8.50%	0	0
	initial RLT	12	25.50%	12	25.50%	0	0
	1st Rechallenge RLT	14	29.80%	13	27.70%	1	2.10%
Anemia	pre-RLT	35	74.50%	33	70.20%	2	4.30%
	initial RLT	40	85.10%	37	78.70%	3	6.40%
	1st Rechallenge RLT	44	93.60%	38	80.50%	6	12.80%
GFR	pre-RLT	31	66.00%	30	63.80%	1	2.10%
	initial RLT	39	83.00%	38	80.90%	1	2.10%
	1st Rechallenge RLT	41	87.20%	40	85.10%	1	2.10%

## Discussion

PSMA-targeted RLT has opened up promising treatment perspectives of mCRPC with remarkable response rates, prolonged survival and a favorable side effect profile in prospective studies [12, 14, 17, 26]. Patients with mCRPC initially benefitting and responding to RLT will, however, experience re-progression at some point. Since therapy options are limited at this stage of disease, a rechallenge approach with recurrent application of RLT appears intuitively reasonable. However, data regarding this approach is still scarce. Addressing this issue, we analyzed the cohort of patients from the prospective registry *(REALITY Study*; NCT04833517*)* who received RLT rechallenge, focusing on response rate, outcome and toxicity. This study demonstrates a high level of efficacy and safety of this rechallenge approach suggesting it indeed as a valuable and promising treatment option.

In this study, we analyzed a cohort of *n* = 47 patients receiving PSMA-RLT rechallenge and observed a substantial response rate of 57.4% and a mean PSA decline of 46.6% in the entire rechallenge cohort. Furthermore, we were able to show that the application of multiple rechallenge series during the course of disease was safe and effective. Our results confirm previous studies of small patient cohorts with RLT rechallenge [21–23]. Violet et al. evaluated 15 patients who were treated with one series of rechallenge [^177^Lu]Lu-PSMA-617 RLT. In this analyzed group 73% of the patients showed a PSA decline ≥ 50%. In a cohort of 30 patients, Yordanova et al. reported a benefit of [^177^Lu]Lu-PSMA-617 RLT rechallenge in 75–90% patients experiencing either stable disease or a response in the first 4 rechallenge cycles. Gafita et al. investigated the feasibility of [^177^Lu]Lu-PSMA-I&T RLT rechallenge in a small cohort of 8 patients. The authors observed that the second treatment series was effective with a response rate of 37.5%. While these authors already reported satisfying responses on a first rechallenge attempt, our study showed that even up to 3 rechallenge series (in total up to 17 cycles) continued to be effective. All patients who responded favorably to a first rechallenge and received multiple rechallenges also benefited from the second (*n* = 10) or even a third (*n* = 3) rechallenge series. This suggests that repeated response can be expected following a first successful rechallenge.

Remarkably, the PFS observed after the first rechallenge RLT (median 8.7 month) was only slightly shorter compared to PFS after initial RLT (median 10.8 month). In addition, for patients responding to rechallenge, a correlation analysis revealed a significant moderate correlation between PFS observed at initial treatment and rechallenge. Specifically, this indicates a relatively long PFS for those patients who showed long PFS on initial RLT.

Similarly promising results were also found for OS. Starting with the application of the first rechallenge a median time of 22.7 mo was observed, a very encouraging result with respect to the advanced stage of disease. Particularly, for patients who achieved a reduction of PSA of at least 50%, a significantly longer OS of 27.3 vs. 10.2 mo for non-responders was observed. The median OS of our cohort is in line with previous studies on RLT rechallenge which reported an OS range between 12 mo and 26.6 mo on smaller cohorts of patients [21–23].

The repeated application of an initially successful therapy is a concept already used in clinical practice, for example in the application of chemotherapy, not only in prostate cancer but in other malignant diseases as well [27–32]. However, in contrast to RLT, chemotherapy is often used in earlier stages of prostate cancer [33, 34]. The reported OS for rechallenge chemotherapy with docetaxel and cabazitaxel ranges between 13.7 and 43.5 mo [35–38]. Differences in stage of disease and applied chemotherapy drugs may explain this wide range. Nevertheless, it should be of note, that the respective studies also report on a variety of side effects caused by rechallenge chemotherapy. In this context, the adverse events of RLT rechallenge must also be taken into account. The safety profile of initial and rechallenge RLT was assessed as favorable, meaning for the majority of patients, that initial treatment and rechallenge had similar limited side effects and was overall well tolerated without treatment termination due to adverse events. In line with our findings, Mader et al. who treated patients in an extended PSMA-RLT therapy concept (comprising up to 16 cycles) observed a comparable safety profile with limited toxicity [39].

In summary, rechallenge PSMA-RLT was effective and safe in this retrospective analysis. We found no counter-argumentation against the concept of rechallenging patients with RLT and considering this as a treatment option for mCRPC patients, who previously benefited from initial PSMA-RLT. To strengthen these conclusions, further research in larger cohorts, preferably in a prospective setting, is clearly recommended. Furthermore, therapy approaches combining rechallenge RLT either with systemic therapy or radiosensitizer and RLT with alpha emitters might also be an option in the future [23, 40–46]. Especially the alpha emitter ^225^Ac, either as monotherapy e.g. [^225^Ac]Ac-PSMA-617 or within a tandem radionuclide concept, e.g. combining [^225^Ac]Ac-PSMA-617 and [^177^Lu]Lu-PSMA-617, has proven to be effective in end-stage mCRPC, especially in patients with insufficient response to monotherapy with ^177^Lu-labeled PSMA ligands [43–46]. These concepts might also be worth an evaluation in a rechallenge setting for patients who have previously responded to the initial series. In addition, the use of other beta-emitting radionuclides such as e.g. ^161^Tb was recently discussed and could be an option for use in rechallenge. These approaches should be investigated and compared in future studies to optimize rechallenge RLT.

Limitations of the present study should be considered which may restrict the interpretation and generalization of the results. First, the study suffers from its retrospective nature and involves only a relatively small number of patients. Second, administered ^177^Lu activity and number of cycles were individually chosen and no standardized protocol was applied. Further studies are needed to investigate the ideal ^177^Lu activity to be used for PSMA-RLT rechallenge. Future studies in larger cohorts should also evaluate predictive parameters to further optimize management strategies for end-stage mCRPC. Due to only intermittently performed PSMA PET/CT imaging and missing contrast-enhanced diagnostic CT scans, we only considered PSA-based PFS and were not able to determine a valid radiological PFS.

## Conclusion

[^177^Lu]Lu-PSMA-617 RLT rechallenge is associated with significant PSA response and encouraging survival outcome as well as a very favourable safety profile. Therefore, this concept of rechallenge RLT should be considered as a treatment option in mCRPC patients, who previously benefited from initial PSMA-RLT. Future studies evaluating [^177^Lu]Lu-PSMA-617 RLT rechallenge in a prospective setting are strongly recommended.

## Electronic supplementary material

Below is the link to the electronic supplementary material.

Supplementary Material 1

## Data Availability

The datasets used and analyzed during the current study are available from the corresponding author on reasonable request.
