# Supplementary material for: Efficacy and safety of rechallenge [177Lu]Lu-PSMA-617 RLT after initial partial remission in patients with mCRPC: evaluation of a prospective registry (REALITY study)
Source: Eur J Nucl Med Mol Imaging. 2024 Jul 15;51(13):4151–62. doi: 10.1007/s00259-024-06825-4 (PMC11527919; doi:10.1007/s00259-024-06825-4)
Supplement: Supplementary file 1 — Supplementary Material 1 [file 259_2024_6825_MOESM1_ESM.docx]

**Supplemental data**

**Table S1** *Detailed information of the initial series of [^177^Lu]Lu-PSMA-617 RLT.*

| **No. of cycles** | **No. of patients** | **Median administered activity/cycle**  (GBq) | **Total administered activity**  (GBq) | **Minimum activity/cycle**  (GBq) | **Maximum activity/cycle** (GBq) |
| --- | --- | --- | --- | --- | --- |
| 1 | 2 | 6.9 | 7.5 ± 0.55 | 6.90 | 8.00 |
| 2 | 8 | 6.2 | 13.5 ± 3.1 | 4.75 | 9.10 |
| 3 | 16 | 6.3 | 18.8 ± 2.9 | 4.33 | 8.13 |
| 4 | 12 | 6.2 | 24.6 ± 4.1 | 4.33 | 8.15 |
| 5 | 4 | 6.5 | 32.8 ± 4.6 | 5.70 | 7.54 |
| ≥6 | 5 | 7.2 | 43.6 ± 4.7 | 5.83 | 7.86 |

**Table S2.** *Patient-based incidence and severity of adverse events (CTCAE) observed in patients receiving two (N=10) or three (N=3) rechallenge series of of [^177^Lu]Lu-PSMA-617 RLT.*

|  |  | Total | Grade 1/2 | Grade3/4 |
| --- | --- | --- | --- | --- |
|  |  | n/N | n | n |
| Any | pre-RLT  initial RLT  1^st^ Rechallenge RLT  2^nd^Rechallenge RLT  3^rd^Rechallenge RLT | 9/10  10/10  10/10  10/10  3/3 | 8  9  9  8  2 | 1  1  1  2  1 |
| Xerostomia | pre-RLT  initial RLT  1^st^ Rechallenge RLT  2^nd^Rechallenge RLT  3^rd^Rechallenge RLT | 0/10  0/10  1/10  1/10  3/3 | 0  0  1  1  3 | 0  0  0  0  0 |
| Fatigue | pre-RLT  initial RLT  1^st^ Rechallenge RLT  2^nd^Rechallenge RLT  3^rd^Rechallenge RLT | 2/10  2/10  2/10  6/10  3/3 | 2  2  2  6  3 | 0  0  0  0  0 |
| Leukopenia | pre-RLT  initial RLT  1^st^ Rechallenge RLT  2^nd^Rechallenge RLT  3^rd^Rechallenge RLT | 3/10  1/10  2/10  3/10  0/3 | 3  1  2  3  0 | 0  0  0  0  0 |
| Thrombo-cytopenia | pre-RLT  initial RLT  1^st^ Rechallenge RLT  2^nd^Rechallenge RLT  3^rd^Rechallenge RLT | 0/10  2/10  1/10  2/10  1/3 | 0  2  1  2  1 | 0  0  0  0  0 |
| Anemia | pre-RLT  initial RLT  1^st^ Rechallenge RLT  2^nd^Rechallenge RLT  3^rd^Rechallenge RLT | 7/10  9/10  9/10  9/10  3/3 | 7  9  9  8  3 | 0  0  0  1  0 |
| GFR | pre-RLT  initial RLT  1^st^ Rechallenge RLT  2^nd^Rechallenge RLT  3^rd^Rechallenge RLT | 7/10  7/10  10/10  9/10  3/3 | 6  6  9  8  2 | 1  1  1  1  1 |

***Figure S1****: Individual course of GFR in patients receiving two (N=10) or three (N=3) rechallenge series of [^177^Lu]Lu-PSMA-617 RLT.*
